# Case report and literature review: Robot-assisted laparoscopic left renal mucinous cystadenocarcinoma radical nephrectomy

**DOI:** 10.3389/fsurg.2022.1053852

**Published:** 2023-01-06

**Authors:** Zikuan Ning, Haoxun Zhang, Bowen Wang, Yingwei Wang, Yiwen Liu, Boju Tao, Guoling Zhang, Hua Liu, Chunyang Wang

**Affiliations:** ^1^ Department of Urology Surgery, The First Affiliated Hospital of Harbin Medical University, Harbin, China; ^2^ Department of Pathology, The First Affiliated Hospital of Harbin Medical University, Harbin, China

**Keywords:** mucinous, cystadenocarcinoma, kidney, primary, robot-assisted surgery

## Abstract

**Background:**

Mucinous cystadenocarcinoma (MC) of the kidney is a rare renal epithelial tumor originating from the renal pelvic urothelium. There are only a few published reports on MC. Due to its rare and unknown tissue origin, its diagnosis is difficult which almost can be diagnosed through the pathological method.

**Case presentation:**

In this case report, we report a female patient whose chief complaint was low back pain lasting for one month. The three-dimensional computed tomography scan of the urinary system detected approximately 7 cm of a left renal cystic mass. The renal cystic mass was diagnosed as MC after robot-assisted laparoscopic radical nephrectomy. The MC originated from the kidney after completing colorectal adenocarcinoma and ovarian adenocarcinoma.

**Conclusions:**

We reported a case of MC of the kidney which was a rare renal tumor. We not only aimed to present an unusual case of MC and review the previous literature on its pathology and differential diagnosis, but also used new method to treat this type of tumor.

## Introduction

The MC of the kidney is a rare tumor that accounts for less than 1% of malignant kidney tumors of the renal pelvis (1). However, its origin from the primary kidney is uncommon. Due to its rarity, it cannot be directly diagnosis in lieu of clinical and imaging features. It presents on computed tomography as a cystic renal mass, and a differential diagnosis mostly results in a misdiagnosis. Here, we reported a rare case of the MC of the primary kidney. This is the first case in which a Da Vinci robot was used to radically resect the left renal MC including the ports placement and operative details. We aimed to report this rare tumor and retrospectively review the pathology, radiology images, and surgery with challenges in cases of misdiagnosis of this type of neoplasm. This case report was reported in agreement with principles of the CARE guidelines (2) and its reporting checklist was as the supplementary material.

## Case report

**Figure 1:**
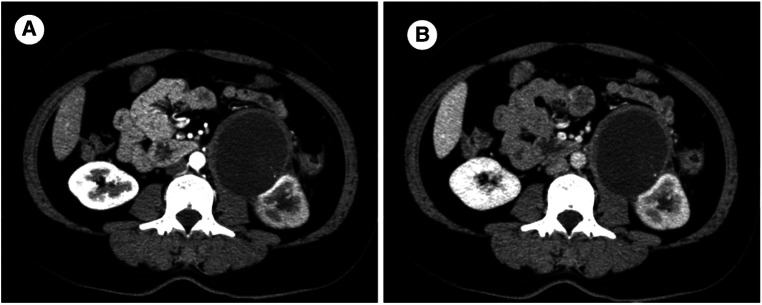
Enhanced computed tomography scan: A round low-denisty cystic mass located at the renal helium, measuring 7.4 × 7.4 cm in size with a width of 0.5 cm at the wall. (**A**) Arterial phase (**B**) Venous phase.

A 46-year-old female patient presented with a complaint of left lower back pain for one month. One month prior, the patient had developed intermittent left lumbago without radiation pain. She had no urinary irritation, hematuria, fever, night sweats, or other uncomfortable feelings. She had a history of transvaginal myomectomy 7 years ago, left lumbar impact injury 9 years ago, and smoking for 10 years. She denied a history of hepatitis, tuberculosis, hypertension, diabetes, and coronary heart disease. There was no percussion pain in the double renal region, and a 6 × 6 cm mass in the left abdomen could be deeply palpated. In additional, there was no tenderness in the ureteral area or edema in either lower limb. An auxiliary examination revealed the following. Three-phase enhanced CT of the kidney showed decreased perfusion of the left kidney and a cystic low-density shadow in the left renal hilum, with a CT value of approximately 5 ± 10 HU and size of 7.4 × 7.4 cm. There were flocculent and high-density shadows in the cyst; however, there was no enhancement in the lesion and slight enhancement in the wall. In addition, the left renal pelvis was dilated, with no abnormal density shadow and a clear perirenal fat space. A small amount of fluid density under the left renal capsule were observed in the images. In general, no significant differences were found in the three-phase enhancement images. No abnormalities were observed in the right kidney. (Figure 1) Pulmonary CT findings were as follows. Nodules were observed under the pleura of the upper lobe of the right lung with a diameter of approximately 3 mm, and a punctate high-density shadow was observed in the upper lobe of the right lung. No abnormalities were found in the abdominal aorta, bilateral iliac artery, bilateral iliac vein, or inferior vena cava. On transvaginal three-dimensional ultrasound, cervical echo was found to be non-uniform. Routine blood examination showed no obvious abnormalities; blood, liver, kidney, and blood coagulation function were normal. The urine routine test results were abnormal: the erythrocyte count was 50.7/μl and leukocyte count was 171.8/μl. After comprehensive analysis, the admission diagnosis included a left renal tumor, left renal hydronephrosis, and left renal subcapsular effusion.

**Figure 2:**
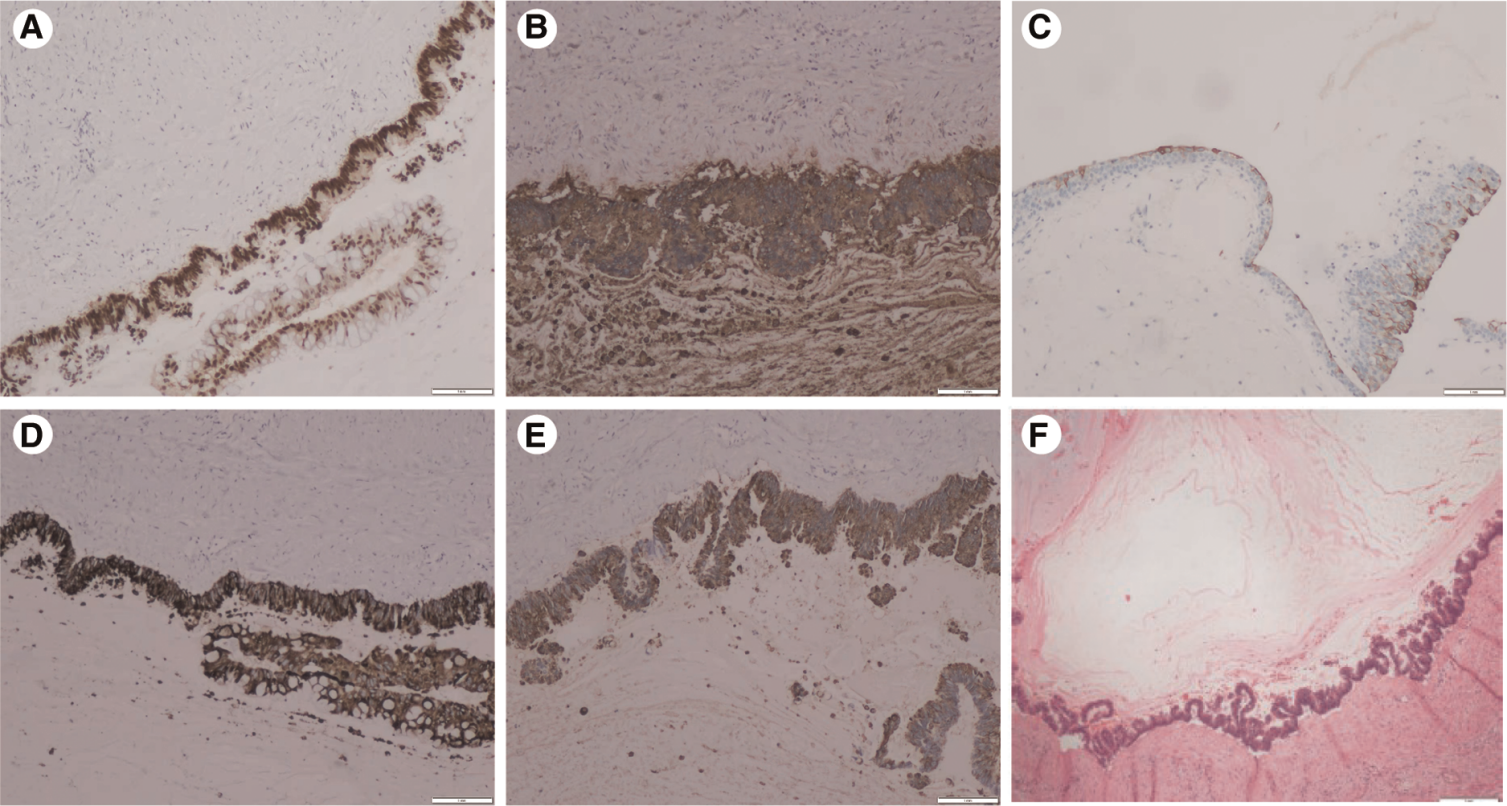
Immunohistochemistry results: (A) CDX2(+): diffuse and strong nuclear immunoreactivity for CDX2. (B) CEA(+): diffuse strong staining for the CEA. (C) CK20(+): atypical mucinous epithelium with patchy cytokeratin 20 staining. (D) MUC2(+) and (E) MUC5AC(+): atypical mucinous epithelium with diffuse strong staining for the MUC2 and MUC5AC gene products (×20) (F) pathology: a wide range of mucus lake was observed under the microscope, and the areas of the cyst wall are lined by atypical mucinous epithelium (H&E staining) (×20).

**Figure 3:**
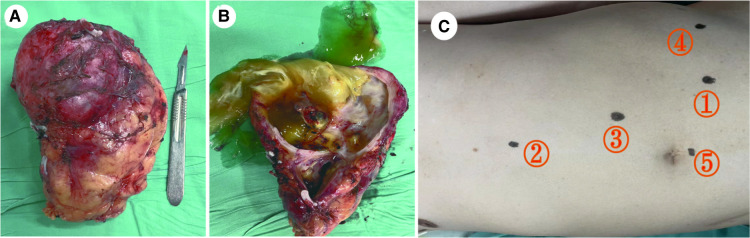
Fresh specimen of the left kidney with mass after radical resection. (A) Gross specimen: The tumor was associated with the kidney and located at the inferior pole. (B) Resection along the vertical axis: The tumor has a large amount of mucus with a focal grayish yellow appearance and thin wall. (C) The ports implacement.

After admission to our hospital, perioperative preparation improved, and robot-assisted radical nephrectomy was performed under combined intravenous infusion and inhalation anesthesia. We used the da Vinci Xi surgical system of the fourth generation to perform the radical nephrectomy. The transperitoneal approach was adopted instead of the traditional retroperitoneal access approach to view the large tumor. The patient was placed in a lying position with the right oblique at 45°. The incision was at the lateral border of the left rectus abdominis under the 3 cm of the umbilical horizontal line. By cutting the subcutaneous tissue of the skin layer by layer, the sheath of the rectus abdominis, and the peritoneum, we put the first 8 mm trocar into the abdominal cavity, which connected to the robot's third arm, placing the monopolar curved scissors, with keeping the pneumoperitoneum pressure at the 14 mmHg level. Laparoscopy was inserted and the other trocars were established under direct vision. The second 8 mm trocar was established at the width of the two fingers under the xiphoid process, which connected to the robot's first arm, placing the maryland forceps bipolar. The third 8 mm trocar was established at the equidistant position between the above two trocars, which connected to the robot's second arm, placing the laparoscopic lens. The area among the trocars and the operative region was kept as an isosceles triangle. Next, the fourth trocar was established at the position of the 2 cm medial to the iliac crest, which connected to the robot's fourth arm, placing a large needle driver. Finally, we established the fifth 10 mm trocar under the umbilicus as an auxiliary port. (Figure 2C) After examination, it was revealed that approximately 10 cm of the round eminence of the splenic curvature of the colon was protruding into the abdominal cavity. After releasing part of the adhesion, the descending colon adhered to the lateral abdomen, and the left peritoneum was cut along the para-colonic groove. The adhesion between kidney and surrounding tissue was severe, and the adhesion around the tumor was also serious, making it difficult to separate. The nature of the tumor was difficult to judge due to wound bleeding, the unclear relationship between between the tumor and kidney, and serious adhesion of the tumor with the psoas major muscle. Because the possibility of malignancy was high, we performed radical resection of the left kidney, including the tumor and part of the ureter. Dissociating the genital vein, and going upstream along it to the renal hilum, then we saw the renal vein. After using Hem-o-Lok clips to disconnect the genital vein, we then separated the renal vein and dissociated the renal artery in the rear. Next, clipping the renal artery and vein with Hem-o-Lok clips, we dissociated the inferior pole of the kidney to sever the ureter. Finally, with preserving the adrenal gland, the left kidney and tumor was removed completely. In the gross specimen, a mass measuring 7 × 7 cm was observed near the hilum of the left kidney and was associated with the ureter. (Figure 2A) After dissection along the vertical axis, there was an outflow of yellowish gelatinous material, and no neoplasm invasion was found in the renal pelvis or ureter. (Figure 2B) During surgery, approximately 800 ml blood was lost; therefore, 400 ml of plasma and 4 U of red blood cells were infused, and no transfusion reaction was observed. Pathology reports revealed that the volume of the left kidney was 11 × 5 × 7 cm, and the volume of the gray-white cystic mass was 7 × 6 × 5 cm at one pole. There was a large amount of mucus in the tumor, which was a focal grayish yellow color, similar to necrosis. There was a local grayish red color around the kidney, and part of the ureter was adherent. The ureter length was 5 cm, and the broken-end diameter was 0.6 cm. Microscopically, a wide range of mucus lakes was observed, in which floating and irregular glands showed large, deep staining of the tumor nucleus and obvious atypia. The image showed mucinous epithelium and a large amount of mucus. (Figure 3F) Immunohistochemical results were as follows: CDX-2(+), Villin(+), CK7(-), CK20(+), CEA(+), MUC2(+), MUC5AC(+), ER(-), PR(-), P16(-), and CA125(-). (Figures 3A–E) Low-grade mucinous adenocarcinoma of the left kidney was pathologically diagnosis; however metastasis was not diagnosed, suggesting the clinical examination of the appendix. No cancer was found at the end of the ureter or vascular end of the renal hilum.

Results of the routine blood examination on the first day after surgery were abnormal: the white blood cell count was 10.5 × 10^9^/L and percentage of neutrophils was 85.9%. The catheter was removed on the second day after surgery. The CT of the upper, middle, lower abdomen was reexamined on the fourth day after surgery. A low-density shadow in the left accessory area, with a small amount of high-density shadow in the pelvic cavity, was observed; furthermore, no abnormalities were found in the other organs. The drainage tube was removed on the fifth day postoperatively, and the patient was discharged without any complaints of discomfort.

Our patient was very satisfied with our surgical method and therapeutic measures. There was no special discomfort after the operation, and she recovered quickly, feeling better than before. She suggested that we deal with her tumor properly and timely.

## Discussion

Primary mucinous adenocarcinoma of the kidney is rare, originating almost entirely from the epithelium of the renal pelvis. Although this type of tumor is located in the renal pelvis, it can also be located in the kidney, ureter, or bladder. To date, approximately 100 cases of mucinous adenocarcinomas of the renal pelvis have been reported. Van Langenhove et al. summarized eight cases of MC of the kidney in the last 10 years after searching the PubMed database (3). We found only one case of MC of the kidney originating from one pole of the kidney without invading the renal pelvis and ureter, which was resected through partial nephrectomy (4). In this paper, we report a case of the MC of the kidney that was located at the lower pole. The disease occurs frequently in men, and there are on specific symptoms, such as mucusuria, flank pain, hematuria, or symptoms that are generally caused by kidney stones and pyelonephritis. In addition, the tumor is generally large, and the abdominal mass is palpable (3, 5). The patient only presented symptoms of left lower back pain. Several theories about the histogenesis of MC in the kidney have been proposed to explain the metaplasia of glands in the pluripotent uroepithelium of the collecting system. The three histogenetic theories include chronic stimulation and differentiation of coelomic epithelial and renal dysplasia (6).

Radiological examinations included ultrasound, CT, or magnetic resonance imaging (MRI) to locate and evaluate the nature of the tumor. However, MC has no unique radiological characteristics. Therefore, distinguishing between benign or malignant tumors or determining the tumor origin are difficult using preoperative radiographic images. After summarizing 30 cases of mucinous adenocarcinomas of the renal pelvis, Li et al. found that mucinous adenocarcinomas presented as multiple renal pelvic calculi, severe hydronephrosis, calculous pyonephrosis, or ureteric junction obstruction, with few cases manifesting as a mass or tumor (5). Although, unique imaging features are lacking to differentiate mucinous adenocarcinomas from common renal tumors, there are also substantial proposals to involve urologists. Most papillary renal cell carcinomas are commonly associated with lymph node metastasis or renal vein infiltration (3). In our imaging findings, because the tumor secreted mucus, it turned into a low-density mass with a slightly thicker and smoother cyst wall on CT, with a CT value of 5 ± 10 HU and flocculent high-density shadow. Enhanced CT suggested no obvious enhancement in the content and slight enhancement in the cystic wall; the tumor could be classified as a Bosniak III. After consulting with general orthopedic surgeons and performing an improved three-dimensional CT scan of the pancreas, an invasive relationship or serious adhesion between the tumor and surrounding tissues, including the psoas major and pancreas, was not found. Because the imaging findings were not specific, we considered the hematoma to be caused by a history of trauma or retroperitoneal tumor. Apart from imaging, special auxiliary examinations may also help in the diagnosis of mucinous adenocarcinomas of the kidney. Some cases have indicated that urine exfoliative cytology might serve as a useful tool when combined with clinical findings. In addition, several studies have also described the important role of CEA and CA199 plasma levels as useful markers before surgery to diagnose mucinous adenocarcinoma and as independent markers for prognosis and recurrence (7).

Renal MC generally have multiple cysts, and the tumor body is large and contains glue-like substances. Most of the cyst walls are smooth, white, or pink, and the local position may have nodular, granular, or micropapillary structures (8). According to the pathological examination results of this patient, a gray-white cystic mass measuring 7 × 6 × 5 cm and a large amount of mucus in the tumor were observed in the lower pole of the kidney, which was closely related to the ureter. Many mucous lakes and mucoid columnar epithelium were observed under the microscope, which appeared highly similar to the cellular epithelium of colorectal adenocarcinomas. Unlike benign adenomas, we found that the tumor cells showed invasive growth microscopically. As stromal invasion is a definitive marker of malignancy, MC was diagnosed.

Considering that the pathological feature of the tumor is the secretion of a large amount of mucus, we analyzed the tumors from a neoteric perspective. The patient was thought to have either a mucinous tubular and spindle cell carcinoma (MTSCC), mixed epithelial and stomal tumor (MEST), or renal abscess. Particularly, MTSCC cells can secret mucus. Fine et al. categorized MTSCC into different subtypes according to the proportions of mucus, tubular, and fusiform components. One subtype is typical, which combines three components, and the other subtype is mucin-lacking, which has little mucus but is full of spindle cells or tubules (9). In addition, the tumor is located in the renal parenchyma with expansive growth and a cystic-solid tumor with a clear boundary as determined by imaging. Plain CT scans are generally isodense or have low density (10). Enhanced CT showed that the density of the tumor was significantly lower than that of the renal cortex and medulla (11). However, in this case, there were only mucous matrix and mucous epithelial cells, no tubular or spindle cells were found, and the imaging was not consistent; therefore, we ruled out the diagnosis.

Furthermore, MEST has a mucous matrix composition. It is a complex cystic and solid mass characterized by the presence of stromal components similar to the stroma of the ovary (composed of fusiform cells with full nuclei and rich cytoplasm) and epithelial component of cysts with an epithelial lining (12). MEST is a complex tumor composed of large cysts, microcapsules, and tubules. The largest cysts consist of columnar and cuboidal epithelia, which occasionally forms small papillary masses. The mucous stroma and fascicular area of the smooth muscle cells may protrude (13). Chu et al. reported a case of a borderline MEST-secreting myxoid matrix (14). The imaging manifestations are not specific. In a typical MEST, an expansive multilocular cystic mass may protrude into the renal pelvis with varying sizes and a thick cystic septum. The tumor does not have a thick fibrous wall, but the cystic septum is thicker than a typical cystic nephroma (13). In our case, there were no special structures observed under the microscope, except for the mucinous epithelium; therefore, MEST was excluded.

Lastly, renal abscesses had similar imaging findings. Renal abscesses are characterized by complex renal cysts with fluid density, uneven intensity, and thick and irregular walls. Owing to the presence of viscous pus, the liquid components show strong and uneven diffusion limitations on diffusion-weighted imaging. Contrast-enhanced CT showed well-defined, low-density round masses, often with thick edges or halos. The abscess had extended around the kidney. MRI showed inhomogeneous thick-edge lesions with low signal intensity on T1-weighted images and high signal intensity on T2-weighted images with limited diffusion (12, 15).

Renal MC can be primary or secondary. However, because primary mucinous adenocarcinoma of the kidney is relatively rare, metastasis from other primary origins, including the pancreas, ovary, colorectum, appendix, should first be excluded. The immunohistochemical results, CDX-2(+), CK20(+), CEA(+), MUC2(+), MUC5AC(+), Villin(+), and CK7(-), were almost similar between the intestinal and ovarian phenotypes. However, combined with the imaging findings of the patient, the origin of the primary lesion could not be determined. Only one case of low-grade cystadenocarcinoma of the appendix presenting as a renal tumor was previously published by Gómez-Román et al. in 1995 (16). The three intestinal tumor markers, CDX2(+), CK20(+), and CK7(-), were similar to the immunophenotype of colorectal adenocarcinoma. Colorectal adenocarcinoma is characterized by CK7(-) and CK20(+) (17). Both the CK7-/CK20 + phenotype and CDX2 antibody expression are highly specific and sensitive markers of the origin of colorectal cancer, and the specificity of CK7-/CK20+ is 97.6% (18). Therefore, it is necessary to consider whether the tumor is of intestinal origin. CDX2 can also be expressed in ovarian mucinous adenocarcinoma, but most express CK7(+) and CK20(-). In addition, MUC2 and MUC5AC are secretory mucins, and the biological significance of their positive expression is mainly in the formation of high-viscosity gel-like mucus. These immunostaining results support another differentiation theory of the histogenesis of the coelomic epithelium in MC in which the peritoneum (mesothelial) undergoes mucinous metaplasia and mucinous cystadenoma (4).

Radical nephrectomy is generally used in the surgical treatment of renal MC; however, Chung et al. reported the use of a partial nephrectomy (4). However, partial resection may be associated with the risk of recurrence and tumor rupture, leading to peritonitis. Some reports have also suggested an additional ureterectomy to improve prognosis, namely conventional nephroureterectomy with a bladder cuff, in case of mucusuria to prevent implantation of tumor cells, due to its development in areas such as the bladder and ureter where the urothelium was present (5). In this case, we performed robot-assisted laparoscopic radical resection of a left renal tumor, and the operation was completed using a transperitoneal approach because the relationship between the tumor and surrounding tissue was unclear and adherent. This was the first robot-assisted laparoscopic radical nephrectomy of MC of the kidney. We considered partial resection of the tumor; however, in view of the situation during the operation and after communicating with the family, we performed robot-assisted laparoscopic radical nephrectomy, and the tumor was completely removed. Compared with laparoscopic radical nephrectomy, robotic radical nephrectomy has obvious advantages, such as providing 3D visual effects with a wider and clearer range of observation and reducing the interference of the instrument, so that the operator can accurately view the anatomy for accurate suture and tremor of the surgeon's hand can be eliminated (19). Autorino et al. Concluded that patients who underwent robotic radical nephrectomy had a higher histological grade and pathological stage and shorter hospital stay, but no difference in operative complications were observed. These results suggest that robotic radical nephrectomy could treat patients with a more advanced and challenging MC (20). Similarly, this case adopted a transperitoneal approach, which is different from the traditional retroperitoneal access approach. The retroperitoneal approach is associated with an earlier recovery of intestinal function, shorter hospital stay, earlier recovery, shorter operation time, and shorter renal hilar vascular control time. However, the transperitoneal approach has a wider workspace and anatomical landmarks and locations can be identified easily (21). Considering the larger tumor size, risk of adhesion with other tissues, and practical needs, we chose transperitoneal access. In summary, we have provided a new method to treat renal MC. According to this case, the challenges of the robotic approach with a mucinous tumor included the fat saponification, severe adhesion with surrounding tissue, and difficult separation. Besides, the cystic renal occupied lesion was easy to be punctured, and leading to peritonitis if the tumor was ruptured. The patient recovered quickly postoperatively. The catheter was removed on the second postoperative day, and the drainage tube was removed on the fifth day after the operation.

**Figure 4:**
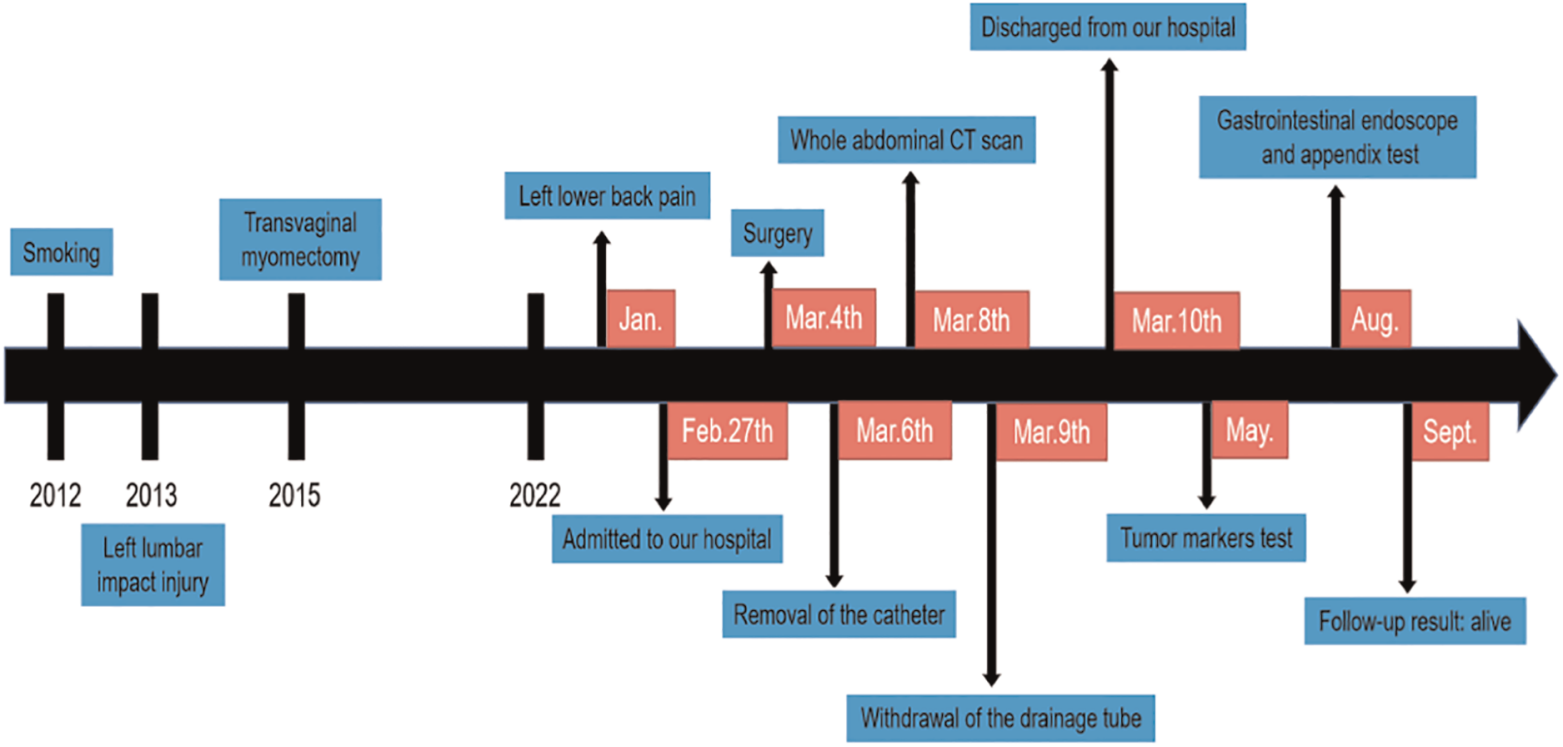
Timeline of the historical and current information from this episode of care.

The prognosis of primary adenocarcinoma of the renal pelvis is generally poor, with most patients dying during the 2–5 year follow-up period. The longest reported follow-up period with good prognosis was 79 months (22). Chemotherapy, radiotherapy, and chemoradiotherapy has been recommended for the treatment of mucinous colorectal cancer and mucinous ovarian carcinoma (5). Therefore, we have adopted adjuvant therapy, such as chemotherapy after surgery, to prolong the survival time of patients. After following-up for half a year, the patient not only recovered well without any discomfort, but also the life and work of the patient were not affected with the weight increasing 10 kg. We did not give the patient adjuvant drug therapy, radiotherapy or chemotherapy. After discharging from our hospital, the patient improved gastrointestinal endoscope and appendix examination, the gastrointestinal tumor and appendix abnormality were not found, and no local recurrence and distant metastasis were found on the whole abdominal CT scan. (Figure 4) We will continue to follow up in future.

## Conclusion

We are the first to report a case of renal MC resected by robot-assisted laparoscopic radical nephrectomy. We have also reported another case in which the tumor was on the kidney without invading the renal pelvis and ureter. MC of the kidney is so rare that is cannot be diagnosed using radiological images; consequently, it can be misdiagnosed to a great extent. Although pathological diagnosis is the gold standard, we still considered the tumor as a local lesion or metastasis of intestinal or ovarian origin. In addition, preoperative examinations, including urine exfoliative cytology and serum levels of CEA and CA199, may improve the efficiency of diagnosis. In this study, we report a tumor that secreted mucus, the origin of the neoplasm, and the advantages of using robotic technology. When encountering a similar patient, we recommend following this situation.

## Data Availability

The original contributions presented in the study are included in the article/Supplementary Material, further inquiries can be directed to the corresponding author/s.
